# Multiscale Modeling of Drug-induced Effects of ReDuNing Injection on Human Disease: From Drug Molecules to Clinical Symptoms of Disease

**DOI:** 10.1038/srep10064

**Published:** 2015-05-14

**Authors:** Fang Luo, Jiangyong Gu, Xinzhuang Zhang, Lirong Chen, Liang Cao, Na Li, Zhenzhong Wang, Wei Xiao, Xiaojie Xu

**Affiliations:** 1 Beijing National Laboratory for Molecular Sciences, State Key Lab of Rare Earth Material Chemistry and Applications, College of Chemistry and Molecular Engineering, Peking University, Beijing, China; 2 National Key Laboratory of Pharmaceutical New Technology for Chinese Medicine, Kanion Pharmaceutical Corporation, Lianyungang, Jiangsu Province, P. R. China

## Abstract

ReDuNing injection (RDN) is a patented traditional Chinese medicine, and the components of it were proven to have antiviral and important anti-inflammatory activities. Several reports showed that RDN had potential effects in the treatment of influenza and pneumonia. Though there were several experimental reports about RDN, the experimental results were not enough and complete due to that it was difficult to predict and verify the effect of RDN for a large number of human diseases. Here we employed multiscale model by integrating molecular docking, network pharmacology and the clinical symptoms information of diseases and explored the interaction mechanism of RDN on human diseases. Meanwhile, we analyzed the relation among the drug molecules, target proteins, biological pathways, human diseases and the clinical symptoms about it. Then we predicted potential active ingredients of RDN, the potential target proteins, the key pathways and related diseases. These attempts may offer several new insights to understand the pharmacological properties of RDN and provide benefit for its new clinical applications and research.

Traditional Chinese medicine (TCM), which mainly consists of herbal medicines, has been practiced over thousands of years. Today it is still regarded as an important source for drug discovery and is growing in popularity worldwide. Therefore understanding the action mechanism of TCM is significant for the treatment of disease. But it is difficult to study the action mechanism of TCM using routine methods due to that herbal formulae are mixtures of lots of chemical ingredients and most of ingredients may interact with multiple targets^1^ weakly or moderately. TCM classified a patient by their ZHENG (TCM syndrome or pattern)^2^. Although the high throughput technologies such as gene expression microarrays^3^, proteomics^4^, and metabolomics^5^ were used to study the mechanisms of TCM, the experiments still have limitations which come from complex nature of TCM. With the rapid progress of bioinformatics, network pharmacology was proposed to be a promising way to understand the mechanism of TCM^8^, and it was successfully applied to analyze the anti-rheumatoid arthritis formulae Qing-Luo-Yin^9^. To date, accumulating evidence suggests that the network pharmacology analysis is a powerful way to study the act mechanisms of herbal formula^0^.

With the advent of big data era, our thinking, technology and methodology are being transformed. Along with the development and application of the Internet information technology, the information of drug molecules, target proteins, biological pathways, diseases, clinical data of diagnosis and treatments have been accumulated dramatically, which generates big data in medical field. The research target was shifted from the “causality inference” to the “correlativity analysis” gradually^1^. The characteristics of TCM information had strong similarity to that of big data^2^. The advent of the big data era provided both opportunities and challenges for TCM such as the new computational methods and technologies^3^. Wang *et al* developed kernel-based methods to integrate drug related omics data sources^4^. The drug-gene-disease coherent subnetwork concept was proposed by Li *et al* to group the biological function related drugs, diseases and genes^5^. The multiscal network pharmacology methods and technologies facilitated the evaluation of effect of drug, disease treatment, disease prediction and prevention, and practice in the treatment in terms of TCM and promoted the further research in drug discovery. With the development of computer science, it is easier and faster to process the big data than before. Computer modeling not only saved the cost in money and time but also improved the efficiency of experiments. The Connectivity Map (CMAP) database contains more than 7,000 expression profiles representing treatments from 1,309 compounds^6^, and it provides a useful tool for TCM when combined with microarray analysis.

ReDuNing injection (RDN) is a patented TCM, which contains the effective components extracted from three herbs^7^, *Artemisia annua* L., *Gardenia jasminoides* E. and *Lonicera japonica* T. The components have been proven that they had potential antiviral, significant anti-inflammatory and immunomodulatory activities^0^. It also showed potential effects in the therapy of influenza^1^ and pneumonia^3^.

Recently, several experimental studies related to the RDN were reported. For example, Tang *et al.* evaluated the protective effects of RDN on lipopolysaccharide-induced acute lung injury in rats and its underlying mechanisms of action^7^. Zhang *et al.* assessed the efficacy and safety of RDN for fever, rash and ulcers in children with mild hand, foot, and mouth disease, and no adverse reactions were observed^4^. Li *et al.* developed a method for screening and analyzing the potential bioactive components from RDN using macrophage cell extraction and ultra-high performance liquid chromatography coupled with mass spectrometry^5^. Zhang *et al.* investigated the effect of RDN in the clinic for upper respiratory tract infections by using molecular docking, network analysis and cell-based assays^6^. Finally, they identified 32 active ingredients and 38 potential targets. Chang *et al.* developed and validated a method for screening and determining the concentration of seven antioxidants of RDN^7^. Yang *et al.* studied the effect of RDN for influenza diseases using a novel systems pharmacology-based strategy^8^. Although the above studies proved that RDN had some important effects in the therapy of several diseases such as influenza and upper respiratory tract infections, the results were still not complete due to that the category of disease was limited, and there were little reports about the effects of RDN for other human diseases such as cancer and inherited metabolic disease. It may be because the mechanisms of these diseases are so complex that it is difficult to study their mechanisms using experimental methods. Therefore, most of the underlying principles of RDN were still unclear and it is necessary to be explored further by using other methods such as computer modeling.

In many ways, drug development is the ultimate multiscale optimization^9^. The importance of multiscale modeling is fittingly recognized by the award of the 2013 Nobel Prize in chemistry. Drug development and preclinical-to-clinical mapping provided an ideal context for multiscale modeling^1^. The case for multiscale modeling approaches to improve the efficiency of drug discovery and development has been made over several years in the past^0^. Here we employed multiscale computer model by integrating molecular docking, network pharmacology and the clinical synopses information of diseases to explore the interaction mechanism of RDN on human disease. By using these methods, we analyzed and predicted potential active ingredients of RDN, the potential target proteins, the key biological pathways, the diseases which related to the pathways, their clinical symptoms and ZHENG. New clinical applications for RDN were also predicted by drug-pathway network. The results of our work attempt to offer new insights to understand the pharmacological properties of RDN and provide benefit for its new clinical application and research.

## Results

### Drug-target and target-pathway network

**Figure 1:**
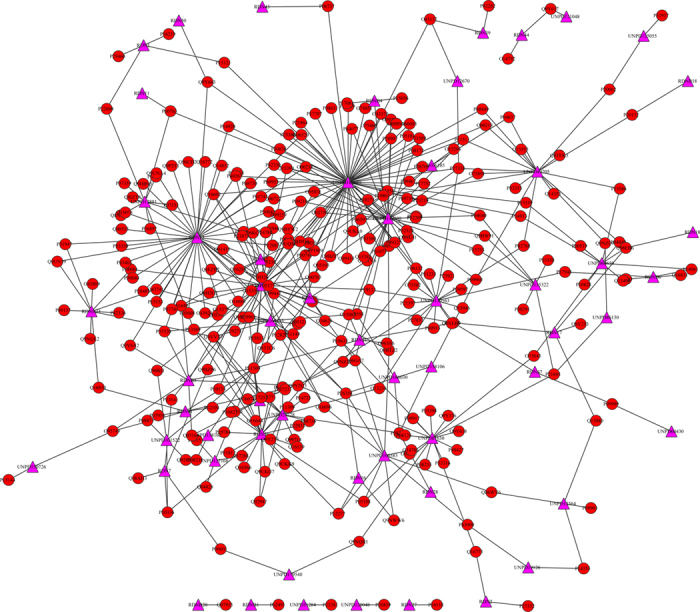
Drug-target network of RDN ingredients and their computational targets. Red circles and pink triangles correspond to target proteins and RDN ingredients, respectively.

We constructed the drug-target network (Fig. 1) based on the docking results, and the network had 333 nodes and 439 edges from Fig. 1, in which red circles and pink triangles correspond to target proteins and RDN ingredients, respectively. Table 1 listed the degree and betweenness of the RDN ingredients molecules. Some molecules have many targets, such as UNPD189689 (62 targets), RDN15 (40 targets) and UNPD149880 (34 targets), which meant that these molecules may be the potential active ingredients of RDN. UNPD189689 is artemisinin, which was also known as *Qinghaosu*, its derivatives were a group of drugs against *Plasmodium falciparum* malaria^1^. Artemisinin was isolated from the plant *Artemisia annua*, which was a kind of herb in Chinese traditional medicine. UNPD149880 is luteolin, it is a plant flavonoid and pharmacologically active agent that had been isolated from the flowers of *Lonicera japonica*^2^ and aromatic flowering plant *Salvia tomentosa* in mint family *Lamiaceae*^3^.

The higher degree of nodes meant highly connected molecules which may play important roles in the drug-target interaction network. RDN15 was a new molecule, which had not the CAS Number, and there were other new molecule that was named by RDN plus number, such as RDNB23. The high degree ingredients of RDN suggested that they could interact with multiple targets and they could have effect on the treatment of multiple diseases. For example, the artemisinin, luteolin and quercetin were proven that they could inhibit LPS-induced PGE_2_ and NO production in experiments^6^. Luteolin had been proven to have anti-carcinogenic^4^, anti-oxidant, anti-inflammatory and anti-allergic activities^5^. In order to explore the possible mechanism of the ingredients of RDN, we classified the ingredient molecules into three parts according their degree in drug-target network: high degree (31-62), middle degree (11-30) and low degree (1-10). Then the interactions between the molecules and target proteins of every part were listed and the relevant pathways were found in KEGG database. At last the score of every relevant pathway was calculated in the three parts (Supplementary Table S0). The score was defined as the relevant frequency of every pathway, which showed the impact of molecules on a certain pathway. Comparing the results of three parts we found that high degree molecules may have more effect on insulin signaling pathway, PI3K-Akt signaling pathway and Tuberculosis than others. Also we analyzed the degree of target protein and found several key target proteins which could interact with most ingredients of RDN. The results was listed in Supplementary Table S1, which showed that P11509 (Cytochrome P450 2A6), Q6VVX0 (Vitamin D 25-hydroxylase) and P19801 (Amiloride-sensitive amine oxidase) could interacted with 12, 7 and 5 molecules, respectively. The first two proteins were related to metabolism system. It showed that the RDN maybe affect the metabolism system of human significantly.

**Figure 2:**
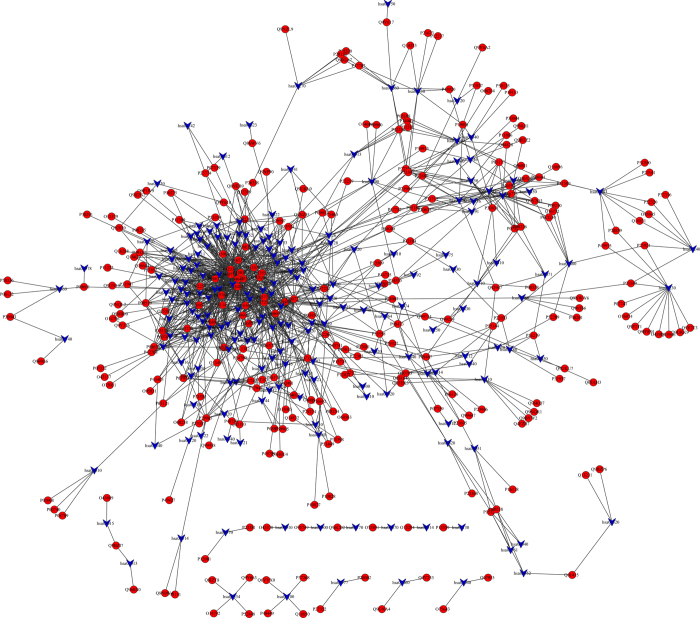
Target-pathway network of RDN ingredients target proteins and their pathways. Red circles and blue V correspond to target proteins and pathways, respectively.

To find the important relations between target protein and the important pathway further, we constructed the target-pathway network (Fig. 2) based on the docking result and the data extracted from KEGG, and the network had 471 nodes and 1252 edges. There were several target proteins in one pathway and one target protein always existed in many pathways. Logically, the role of one pathway which contain many target proteins that interacted with drug molecules is more vital than the role of one target protein that interacted with drug molecules in many pathways, which is because that the impact of one target protein on the whole pathway maybe little, and the impact of a pathway which contained many target proteins interacted with the drugs on the body could be huge. Therefore, we tried to find the important pathways through analyzing the target –pathway network. In Fig. 2, the degree of the pathway showed that the number of the target protein in the pathway. The higher degree of the pathway, the more target proteins existed in the pathway. The parameters of target-pathway network were shown in Table 2.

From Table 2 we found that the pathways with higher degree were neurotrophin signaling pathway, PI3K-Akt signaling pathway, MAPK signaling pathway and focal adhesion, and their degree were 21, 20, 19 and 17, respectively. It meant that there were 21, 20, 19 and 17 target proteins in these pathways could interact with the ingredients of RDN, which suggested that they were the key pathways in the interaction mechanism of RDN. Neurotrophin signaling pathway is regulated by connecting a variety of intracellular signaling cascades, which included PI3K-Akt signaling pathway and MAPK signaling pathway. Also there were several pathways which were classified in metabolism, such as metabolism of xenobiotics by cytochrome P450 and drug metabolism-cytochrome P450.

### Drug-pathway and pathway-disease network

Drug-pathway interactions is very significant not only for understanding the various drug response and molecular interaction process, but also for the development of Chinese traditional medicine and the therapy of human diseases. However, many drugs play a role by affecting all kinds of pathways in the body, such as metabolism pathway or signal transduction pathway. At the same time the effect of drugs to the pathway could affect the diseases that related with the pathways. Building a drug-pathway network would be a direct method to find the relations between the drugs and pathways.

**Figure 3:**
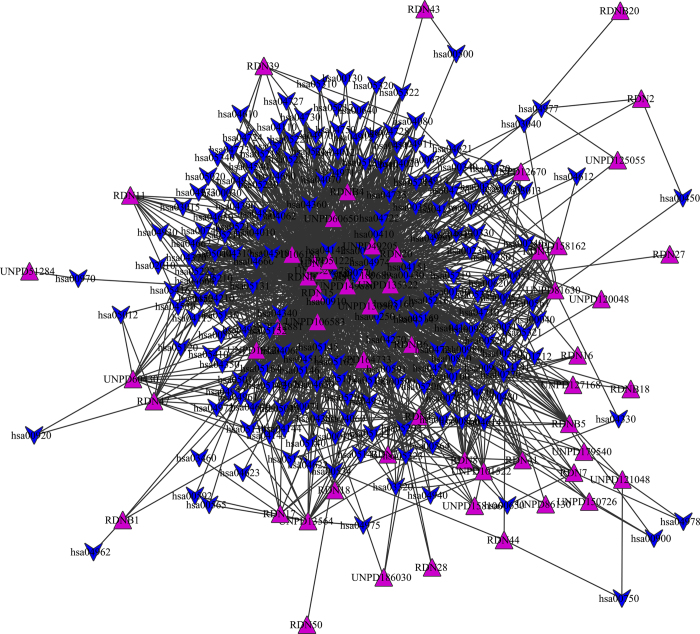
Drug-pathway network of RDN. Representations of the symbols are the same as previous figures.

The drug-pathway network (Fig. 3) was constructed based on the previous networks through connecting the previous two networks by the same target protein, and we predicted potential drug-pathway interactions and the key pathways in the drug interaction of RDN. In Fig. 3, it is obvious that some drugs could interact with multiple pathways and others could only interact with two or three pathways.

The parameters of the drug-pathway network were shown in Table 3. From Table 3 we found that the degree of one molecule was more than that of one pathway. For example, the UNPD189689 could interact with 130 pathways and UNPD149880 (luteolin) could interact with 100 pathways, which showed that these molecules were potential active. This result was consistent with the previous predicted result in the drug-target network. Some pathways were related to cancer such as hsa05210, colorectal cancer pathway, and there were 16 molecules could interact with the proteins of colorectal cancer pathway. It meant that these molecules were potential active for cancer. Experimental results show that luteolin had potential role for cancer prevention and therapy^6^. There were 22 drugs could interact with hsa00982 and 21 drugs could interact with hsa00980. Hsa00982 was Drug metabolism - cytochrome P450 and hsa00980 was metabolism of xenobiotics by cytochrome P450, which was also regarded as an important pathway in the previous networks. Then we selected the pathways which had the higher degree to study further.

**Figure 4:**
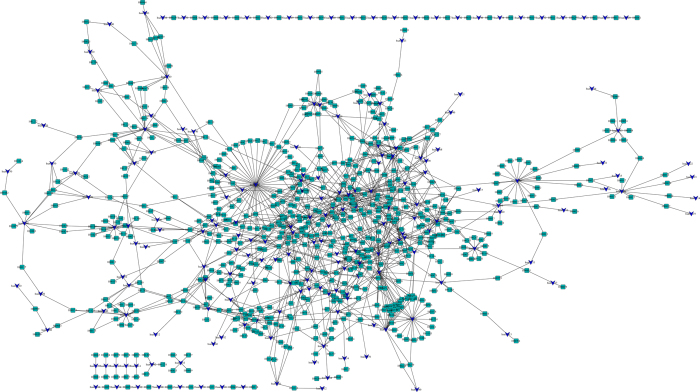
Pathway-disease network of key pathway. Blue V and Cyan round rectangle correspond to pathways and diseases, respectively.

The pathways had often related with some diseases, while the drugs interacted with the pathways, they would affect the related diseases. In order to find the diseases that could be related with the pathways, we constructed the pathway-disease network based on all of pathways to find several key diseases to study further. The pathway-disease network was shown in Fig. 4. The results showed that several pathways were related to only one disease, and other pathways were related to several diseases. Most pathways were related to two diseases at least. On average, a disease is associated with 0.3 pathways and a pathway is associated with 3 diseases. The obvious three larger circles in Fig. 4 were pathways hsa04142, hsa04610 and hsa05202, and were related to 47, 21 and 20 diseases, respectively.

Table 4 listed the key pathways and the related disease. The key pathways were extracted according to their degree in the drug-pathway network, and several high degree pathways belong to the metabolism, such as drug metabolism and retinol metabolism. Other pathways belong to signal transduction, such as MAPK signaling pathway and PI3K-Akt signaling pathway. There were also some disease pathways, for example, diabetes, tuberculosis and influenza A. The molecules of RDN would affect the diseases through the related pathways. And there were several experimental results showed that RDN had effective treatment for the influenza^1^, lung injury^7^ and fever^7^, luteolin of RDN had also been proven to be potential for diabetes^9^, which suggested that our results is reliable.

### Diseases associated with RDN, clinical symptoms and ZHENG

Then we analyzed the category of all of the related diseases. The results were listed in Table 5. We found that the inherited metabolic disease, cancer and nervous system disease were the main disease category, and the proportion of them were 10.73%, 6.11% and 4.62%, respectively. The proportion of other category diseases such as hematologic disease, skeletal dysplasia and infectious disease were 4.02%, 3.43% and 3.13%. Though the proportion of infectious disease is not high, there were several experimental results reported that the RDN was effect on the treatment of some infectious diseases, such as influenza^1^ etc. And the experimental reports of inherited metabolic disease, cancer and nervous system disease were seldom, it may be due to the complex mechanism of those diseases and the different ratio of the ingredients in RDN.

In the post-genomic era, the elucidation of the relationship between the diseases and their clinical phenotypes (such as clinical symptoms of disease and TCM ZHENG) is vital for medical research. An important available resource is symptom, the highest level clinical phenotype, which had often been overlooked in research^0^. To elucidate the relations between the diseases and their clinical symptoms, we collected the symptoms of the diseases (Table 5), which were related to the key pathways of the drug-pathway network through searching the OMIM database and MedlinePlus database. The number of seizures and were 197, which show that there were 197 diseases associated with seizures and the RDN may have effect on nervous system disease. And most of the related diseases were inherited metabolic disease and nervous system disease. Though the number of fever was 46, RDN is proven to be effective for the fever in the clinical experimental results^4^.

**Figure 5:**
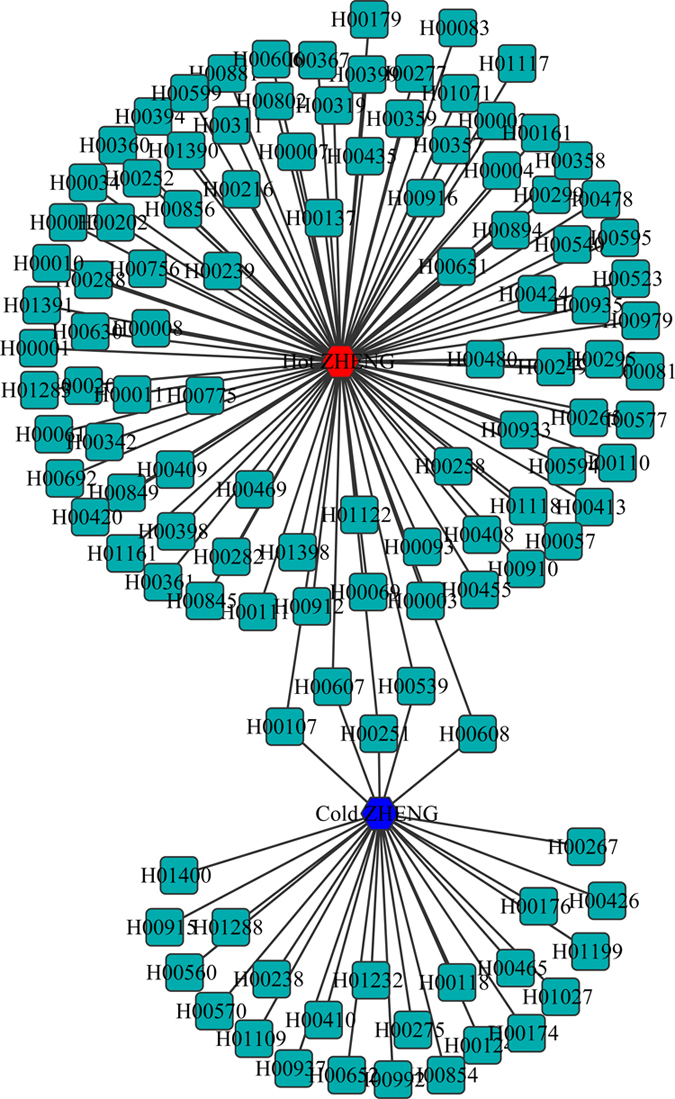
Disease-ZHENG network of RDN. Red and blue hexagon represented the Hot ZHENG and Cold ZHENG, respectively. Other representations of the symbols are the same as previous figures.

ZHENG is the key concept and the basic unit in TCM theory, it has been used for thousands of years in China^1^. ZHENG is not simple assemblage of disease symptoms, it can be regarded as the TCM theoretical abstraction of the symptom profiles of disease. ZHENG had been investigated by using a systems biology approach with the combination of computational analysis and animal experiment^1^. Li *et al.* explored new modalities for the clinical characterization of ZHENG using various supervised machine learning algorithms^2^. Based on the ZHENG-related disease data sets, the 117 diseases associated with ZHENG was found and classified into Cold ZHENG and Hot ZHENG (Fig. 5). We found that there are 28 diseases belong to Cold ZHENG (such as Fanconi anemia), 94 diseases belong to Hot ZHENG (Typhoid fever and influenza) and 5 diseases belong to both Cold ZHENG and Hot ZHENG (PTEN hamartoma tumor syndrome). Table 6 listed the relation between several diseases and ZHENG. The number of ZHENG show that most diseases belong to Hot ZHENG and RDN may have better effects on the treatment of Hot ZHENG such as cancer, fever, influenza and diabetes, and the results was in agreement with the clinical antipyretic and antidotal effect of RDN.

In order to clarify the possible interaction mechanism of RDN, the pathways, target proteins and the possible active ingredients of RDN were analyzed. Here Tuberculosis and influenza were selected as examples to explore the interacted mechanism.

### Tuberculosis

**Figure 6:**
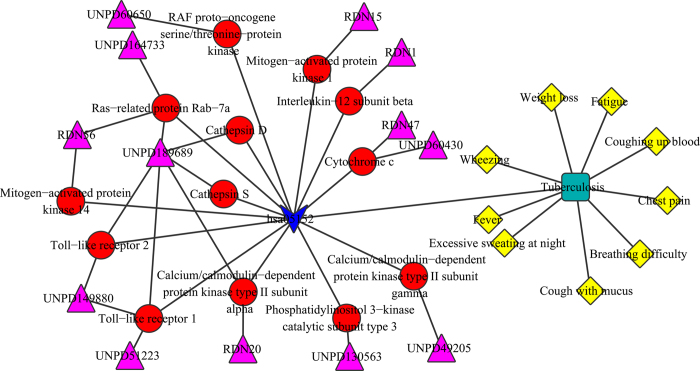
Possible interacted mechanism of RDN ingredients for tuberculosis. Yellow diamond represented the clinical symptoms and other representations of the symbols are the same as previous figures.

Tuberculosis is a widespread infectious disease caused by various strains of mycobacteria, usually *Mycobacterium tuberculosis,* it typically attacks the lungs, but can also affect other parts of the body^3^. Here we predicted the possible mechanism of anti-inflammatory effects of RDN for tuberculosis (Fig. 6). The molecules, target proteins in the pathway of hsa05152 (Tuberculosis) were extracted from the drug-target network and target-pathway network. In Fig. 6, there were 13 molecules (UNPD149880, UNPD189689, UNPD60650 and UNPD49205 etc.) interacted with 13 target proteins in the tuberculosis pathway. The 13 ingredients maybe the main active ingredients and the 13 target proteins were key protein in tuberculosis pathway.

Tang *et al.* had studied the anti-inflammatory effects of RDN on lipopolysaccharide-induced acute lung injury of rats^7^, they found that the anti-inflammatory effects of RDN was demonstrated to be preventing pulmonary neutrophil infiltration, lowering myeloperoxidase activity, TNF-α and iNOS gene expression by inhibiting NF-κB activity in LPS-induced acute lung injury. Our results showed that the target proteins in the pathway were Toll-like receptor 2 (TLR2), RAF proto-oncogene serine/threonine-protein kinase (Raf1), Cathepsin D (CathepsinD), Cathepsin S (CathepsinS), Mitogen-activated protein kinase 1 (ERK1/2), Interleukin-12 subunit beta (IL-12), Ras-related protein Rab-7a (Rab7), Cytochrome c (CytC), Calcium/calmodulin-dependent protein kinase type II subunit gamma (CAMKII), Toll-like receptor 1 (TLR1/6), Mitogen-activated protein kinase 14 (p38), Phosphatidylinositol 3-kinase catalytic subunit type 3 (VPS34) and Calcium/calmodulin-dependent protein kinase type II subunit alpha (CAMKII). The 13 molecules of RDN could affect tuberculosis through interacting with these target proteins in tuberculosis pathway. At the same time, the molecules might have the effect on the symptoms of tuberculosis such as fever and chest pain. The results were different from experimental results of Tang, it may due to the two reason: on the one hand, there are essential differences between the experiment of lipopolysaccharide-induced acute lung injury of rats and tuberculosis of human, on the other hand, our results were based on the docking results, and the targets that have no crystal structure were not contained in our results, which would not limit the experimental study. All the same, our results provided possible mechanism and some insights for the treatment of tuberculosis.

### Influenza

**Figure 7:**
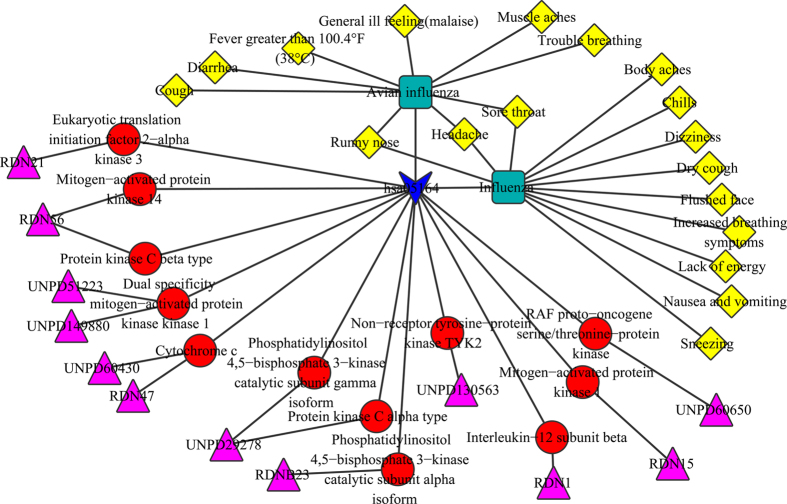
Possible interacted mechanism of RDN ingredients for influenza. Representations of the symbols are the same as previous figures.

Influenza is an infectious disease of birds and mammals caused by RNA viruses of the family Orthomyxoviridae, the influenza viruses. Although it is often confused with the common cold, influenza is a severe disease^4^. According the previous network, the possible interacted mechanism of RDN network was extracted for the influenza (Fig. 7). And there were 12 ingredients including UNPD149880, UNPD60650 interacted with 12 target proteins such as Interleukin-12 subunit beta (IL12) and Phosphatidylinositol 4,5-bisphosphate 3-kinase catalytic subunit alpha isoform (PI3K) in hsa05164. The 12 target proteins were RAF proto-oncogene serine/threonine-protein kinase (Raf), Protein kinase C beta type (PKCβII), Protein kinase C alpha type (PKCα), Mitogen-activated protein kinase 1 (ERK1/2), Interleukin-12 subunit beta (IL12), Non-receptor tyrosine-protein kinase TYK2 (Tyk2/Jtk1), Phosphatidylinositol 4,5-bisphosphate 3-kinase catalytic subunit alpha isoform (PI3K), Phosphatidylinositol 4,5-bisphosphate 3-kinase catalytic subunit gamma isoform (PI3K), Cytochrome c (CytC), Dual specificity mitogen-activated protein kinase kinase 1 (MEK1/2), Mitogen-activated protein kinase 14 (p38) and Eukaryotic translation initiation factor 2-alpha kinase 3 (PKR).

Tang *et al.* investigated the protective effect of RDN against influenza^5^, and their results showed that the effects of RDN for influenza were about to nuclear factor-kappa B (NF-κB) protein and interleukin-1 (IL-1β). Zhou *et al.* suggested that the symptom-based similarity of two diseases correlates strongly with the number of shared genetic associations and the extent to which their associated proteins interact^0^. And we found that there were the same target proteins in the tuberculosis pathway and influenza pathway, such as Raf1, CytC, ERK1/2, IL12 and p38. There were also the same symptoms in tuberculosis and influenza such as cough and fever, so these symptoms may be related to the common target proteins, which was consistent with the results of Zhou. Especially, IL-12 is involved in the differentiation of naive T cells into TH1 cells^6^. It played an important role in the activities of natural killer cells and T lymphocytes, and it can stimulate the growth and function of T cells. The endogenous IL-12 plays an important role in the normal host defense against infection by a variety of intracellular pathogens, and IL-12 appears also to play a central role in the genesis of some forms of immunopathology^7^.

### Comparison of gene expression profiles with the CMAP reference database

The compounds in CMAP reference database were tested only in three human cancer cell lines and not every compound was tested in all cell lines. To further evaluate the quality and usability of our predicted results, we used the gene expression profiles of MCF-7 cells treated with luteolin in CMAP as a query to search the CMAP database. If our data are in good quality and the CMAP approach works, we should find the drug that has an effect on MCF-7 cells similar to that of luteolin. The drug might be performing activities based on same or similar targets, pathways and mechanisms and has similar activities. The query results were listed in Table 7. The high positive score indicates that the corresponding perturbagen in the CMAP database may similarly induce the expression change as the query agent. The mean score of luteolin, apigenin and chrysin were 0.793, 0.722 and 0.573, respectively, which indicated that the latter two drugs have an effect on MCF-7 cells similar to that of luteolin. Their activities might be based on same or similar targets and pathways. Apigenin had been proven to have antiarthritic^8^ and anticancer^9^ activity, chrysin had also been proven to have anti-inflammatory and anticancer^0^ activity. All of these facts show that our results of RDN were reliable.

## Discussion

Though Traditional Chinese medicines had exhibited pharmacologic effects through a large number of clinical practices over thousand years, there were many unclear underlying mechanisms. Multiscale computer modeling provided an effective way to help us to explore the underlying mechanisms of TCM. Here we explored the interaction mechanism of RDN and predicted possible target proteins by employing multiscale computer modeling integrating molecular docking, network pharmacology and the clinical symptoms information of diseases. We constructed the drug-target network, target-pathway network, drug-pathway network and pathway-disease network and found several potential active molecules in RDN and several important interaction pathways. Meanwhile, we analyzed the relations among the drug ingredients, targets, pathways, diseases and clinical symptoms and explored the possible drug interaction mechanism of RDN. For example, we predicted the possible interaction mechanism of RDN ingredients for tuberculosis and influenza. The results of our work attempt to offer new insights to understand the pharmacological properties of RDN and provide benefit for its new clinical applications and research.

## Methods

### Preparation of data sets and molecular docking

The 3D structures of 90 ingredients from RDN were collected from UNPD database^1^ and literature^2^, the crystal or NMR structures of 2715 target proteins were downloaded from Protein Data Bank^3^ according to criteria described in our previous work^4^. The target proteins were used to screen potential active ingredients of RDN by molecular docking. The original ligands of complexes were used to determine the active site and as reference compounds to compare the binding affinity of RDN ingredients to the target proteins. Molecular docking was then carried out by AutoDock 4.0^5^ and DOVIS 2.0^6^ according to the protocol described in our previous work^7^.

To keep the accuracy of predicted results and integrity of data, we selected the molecules which docking scores were higher than the score of original ligand in the complex structure and higher than 6.0, then the target proteins which satisfying the following criteria were selected to construct the drug-target network: (a) The source organism is human. (b) The protein has a KEGG annotation of pathway in KEGG database^8^. (c) The pathway has related disease annotation in KEGG database. Finally, we obtained 57 RDN ingredients and 271 target proteins. The target proteins were associated with 200 human related pathways and 668 diseases extracted from the KEGG database.

### Drug-target network and target-pathway network construction based on docking results

To find the potential active molecules and target protein, the drug-target network (Fig. 1) was constructed according the docking results in Cytoscape^9^. We also calculated the node centralities and network properties using the network analyzer tool of Cytoscape. Then we constructed target-pathway network to find the relation between the pathways and the target proteins. We analyzed the network properties of the target-pathway network and found several important pathways, which could play an important role in the RDN interaction mechanism.

### Drug-pathway and pathway-disease network construction

Many drugs play a role by affecting the related pathways or affecting the metabolisms in the body. Moreover, the state of disease is often the results of a series of variations in our body such as the variations in pathways, instead of the anomaly of drug-target interaction^0^. Therefore, clarifying the mechanism of drug-pathway associations is a crucial issue of system-based drug discovery. To understanding the action mechanism of the drugs we constructed the drug-pathway network based the previous drug-target network and target-pathway network. And then we predicted potential drug-pathway interactions. Several significant pathways were found through analyzing the drug-pathway network. Every pathway corresponded one or more than one diseases, therefore, to illuminate the associations between pathways and the related diseases, we constructed the pathway-disease network based on the data extracted from KEGG database. The pathway-disease relationships were investigated based on the pathways. One pathway maybe related to many diseases and one disease could be related to many pathways. Several diseases shared the common pathways were found through analyzing the parameters of the network.

### Diseases, clinical symptoms and ZHENG

The abnormal of every pathway could result to one or more diseases, and every disease is related to several clinical symptoms. So symptoms play important role in modern medical diagnosis and disease classification, patients with different disease would often present different symptoms. Understanding the underlying molecular mechanisms of symptoms could help us treat disease efficiently. Several symptoms such as depression, pain, and high blood pressure have been discussed previously^3^. Recently, Zhou *et al.* used large-scale biomedical literature database to construct a symptom-based human disease network and investigate the connection between clinical manifestations of diseases and their underlying molecular interactions^0^. The relations between diseases and symptoms can further be used as a resource helping to clarify important questions in the field of systems medicine. In order to explore the relation between the disease and associated symptoms further, the 12664 symptoms associated with 668 diseases were extracted from Online Mendelian Inheritance in Man (OMIM) database ( http://omim.org/) and MedlinePlus database ( http://www.nlm.nih.gov/medlineplus/). Then we selected the diseases associated with ZHENG and classified them into Cold ZHENG and Hot ZHENG according to the ZHENG-related disease and symptom data sets in the work^1^ of Li *et al.* We expected to find the underlying molecular interactions of the symptoms and ZHENG. Finally, we predicted the possible RDN molecular mechanisms of some diseases, and our results were proven by several experimental reports, which showed that the methods we used were reliable.

### Comparison with the CMAP reference database

To further evaluate the quality of the predicted results, we used the gene expression profiles of luteolin as queries to search the “Connectivity Map” (CMAP, http://www.broadinstitute.org/cmap/) reference database (Build 02), which contains more than 7,000 expression profiles mainly from three cell lines (MCF-7, HL60 and PC3) treated with 1,309 compounds^6^. The high positive connectivity score indicates that the corresponding perturbagen in the database may induce the expression change as the query agent similarly. Each combination of chemical names and cell lines has a mean connectivity score and a permutation p value, which shows the probability of enrichment of a set of instances in a list of all instances by chance.

## Author Contributions

X.J.X., W.X. and L.R.C. conceived the study. F.L. and J.Y.G. designed the experiments. F.L. and J.Y.G. performed the experiments with the help of X.Z.Z., L.C., N.L. and Z.Z.W., F.L. and J.Y.G. analyzed the data and wrote the manuscript. All authors reviewed the manuscript.

## Additional Information

**How to cite this article**: Luo, F. *et al.* Multiscale Modeling of Drug-induced Effects of ReDuNing Injection on Human Disease: From Drug Molecules to Clinical Symptoms of Disease. *Sci. Rep.* **5,** 10064; doi: 10.1038/srep10064 (2015).

## Supplementary Material

Supplementary Information

## Figures and Tables

**Table 1:** Chemical information and network parameters of the ingredients in RDN.

**Molecule ID**	**Name**	**CAS NO.**	**Chinese Name**	**Degree**	**Betweenness**
UNPD189689	Artemisinin	63968-64-9	*Qinghaosu*	62	0.434157
RDN15	2-carboxy-4-methyl-α-methylene -3-(3-oxobutyl)-cyclohexaneacetic acid	N/A	N/A	40	0.199621
UNPD149880	Luteolin	491-70-3	*Muxicaosu*	34	0.166964
UNPD51223	Cynaroside	5373-11-5	*Muxicaogan*	32	0.151816
RDN56	Sacranoside B	152520-94-0	N/A	22	0.132405
UNPD49205	Quercetin	117-39-5	*Hupisu*	18	0.099038
UNPD130563	Scoparin	301-16-6	*Jingquehuasu*	16	0.090562
UNPD81630	Vogeloside	60077-47-6	*Duanma qianzi ganbansuo quanneizhi*	16	0.102913
UNPD164733	Cryptochlorogenic acid	905-99-7	*Yinluyuansuan*	14	0.058415
UNPD29278	Lonicerin	25694-72-8	*Rendonggan*	12	0.056157
RDNB23	2’-*O-trans*-caffeoylgardoside	N/A	N/A	10	0.036748

**Table 2:** The network parameters of the target-pathway network.

**Pathway ID**	**Pathway Name**	**Pathway Class**	**Degree**	**Betweenness**
hsa04722	Neurotrophin signaling pathway	Organismal Systems; Nervous system	21	0.019096
hsa04151	PI3K-Akt signaling pathway	Environmental Information Processing; Signal transduction	20	0.056881
hsa04010	MAPK signaling pathway	Environmental Information Processing; Signal transduction	19	0.014711
hsa04510	Focal adhesion	Cellular Processes; Cellular commiunity	17	0.014727
hsa04910	Insulin signaling pathway	Organismal Systems; Endocrine system	17	0.077733
hsa00980	Metabolism of xenobiotics by cytochrome P450	Metabolism; Xenobiotics biodegradation and metabolism	16	0.035646
hsa04370	VEGF signaling pathway	Environmental Information Processing; Signal transduction	16	0.012222
hsa00982	Drug metabolism - cytochrome P450	Metabolism; Xenobiotics biodegradation and metabolism	15	0.047763
hsa04062	Chemokine signaling pathway	Organismal Systems; Immune system	15	0.008937
hsa04726	Serotonergic synapse	Organismal Systems; Nervous system	15	0.104612
hsa04810	Regulation of actin cytoskeleton	Cellular Processes; Cell motility	15	0.007014

**Table 3:** The network parameters of the drug-pathway network.

**Coumpound ID**	**Degree**	**Betweenness**	**Pathway ID**	**Pathway Name**	**Degree**	**Betweenness**
UNPD189689	130	0.235865	hsa00982	Drug metabolism - cytochrome P450	22	0.015074
RDN15	102	0.132960	hsa00980	Metabolism of xenobiotics by cytochrome P450	21	0.013131
UNPD149880	100	0.101266	hsa00830	Retinol metabolism	19	0.010414
RDN56	98	0.094563	hsa00983	Drug metabolism - other enzymes	19	0.016943
UNPD51223	94	0.091357	hsa05206	MicroRNAs in cancer	18	0.021116
UNPD29278	90	0.069452	hsa04010	MAPK signaling pathway	17	0.005958
UNPD49205	68	0.057346	hsa04151	PI3K-Akt signaling pathway	17	0.009336
RDNB23	66	0.031968	hsa04510	Focal adhesion	16	0.005325
UNPD60650	57	0.034424	hsa04722	Neurotrophin signaling pathway	16	0.005382
UNPD130563	47	0.021560	hsa05210	Colorectal cancer	16	0.007149
UNPD106583	42	0.019696	hsa00140	Steroid hormone biosynthesis	15	0.006570
UNPD12881	41	0.021428	hsa05152	Tuberculosis	13	0.005072
UNPD19126	36	0.023967	hsa05164	Influenza A	12	0.003806
UNPD106185	33	0.018818	hsa05132	Salmonella infection	8	0.001548

**Table 4:** the key pathways and the related diseases extracted from pathway-disease network.

**Pathway ID**	**Pathway Name**	**Disease ID**	**Disease Name**
hsa00982	Drug metabolism - cytochrome P450	H01234	Fish odour syndrome
hsa00980	Metabolism of xenobiotics by cytochrome P450	H01203	Primary congenital glaucoma
hsa00830	Retinol metabolism	H00825	Familial flecked retina syndrome
hsa00983	Drug metabolism - other enzymes	H00199	Dihydropyrimidinase deficiency
hsa05206	MicroRNAs in cancer	H00013	Small cell lung cancer
hsa04010	MAPK signaling pathway	H00882	Cocoon syndrome
hsa04151	PI3K-Akt signaling pathway	H01340	Bethlem myopathy
hsa04510	Focal adhesion	H00576	Pierson syndrome
hsa04722	Neurotrophin signaling pathway	H00408	Type I diabetes mellitus
hsa05210	Colorectal cancer	H00020	Colorectal cancer
hsa00140	Steroid hormone biosynthesis	H00134	X-linked ichthyosis
hsa05152	Tuberculosis	H00342	Tuberculosis
hsa05164	Influenza A	H00398	Influenza
hsa05132	Salmonella infection	H00111	Typhoid fever

**Table 5:** Category of the related diseases and the distribution of the clinical synopses.

**Disease Category**	**Percentage**	**Clinical Symptoms**	**Number**
Inherited metabolic disease	10.73	Seizures	197
Cancer	6.11	Mental retardation	183
Nervous system disease	4.62	Hypotonia	150
Developmental disorder	4.47	Developmental delay	94
Hematologic disease	4.02	Hearing loss	86
Skeletal dysplasia	3.43	Vomiting	74
Infectious disease	3.13	Diarrhea	66
Skin and connective tissue disease	3.13	Cataracts	56
Primary immunodeficiency	2.98	Fever	46
Immune system disease	2.68	Fatigue	36
Neurodegenerative disease	1.79	Hypoglycemia	36
Endocrine disease	1.49	Headache	21
Kidney disease	1.49	Cough	20

**Table 6:** The relationship between several diseases and ZHENG.

**Disease ID**	**Disease Name**	**Disease category**	**ZHENG**
H00630	Disease_Rheumatoid arthritis	Autoimmune disease	Hot ZHENG
H00001	Disease_Acute lymphoblastic leukemia (ALL) (precursor B lymphoblastic leukemia)	Cancer	Hot ZHENG
H00013	Disease_Small cell lung cancer	Cancer	Hot ZHENG
H00020	Disease_Colorectal cancer	Cancer	Hot ZHENG
H00539	Disease_PTEN hamartoma tumor syndrome (PHTS)	Developmental disorder; Cancer	Cold and Hot ZHENG
H00251	Disease_Thyroid dyshormonogenesis	Endocrine disease	Cold and Hot ZHENG
H01027	Disease_Anophthalmia and microphthalmia (A/M)	Eye disease	Cold ZHENG
H00238	Disease_Fanconi anemia	Hematologic disease	Cold ZHENG
H00288	Disease_Familial Mediterranean fever (FMF)	Immune system disease	Hot ZHENG
H00111	Disease_Typhoid fever	Infectious disease	Hot ZHENG
H00342	Disease_Tuberculosis	Infectious disease	Hot ZHENG
H00361	Disease_Malaria	Infectious disease	Hot ZHENG
H00398	Disease_Influenza	Infectious disease	Hot ZHENG
H00399	Disease_Avian influenza	Infectious disease	Hot ZHENG
H01071	Disease_Acute alcohol sensitivity	Inherited metabolic disease	Hot ZHENG
H00409	Disease_Type II diabetes mellitus	Metabolic disease; Endocrine disease	Hot ZHENG
H00408	Disease_Type I diabetes mellitus	Metabolic disease; Immune system disease; Endocrine disease	Hot ZHENG

**Table 7:** Top CMAP hits correlated with luteolin treatment.

**Treatment**	**CMap chemical name and cell line**	**Mean score**	**p-value**
Luteolin	Luteolin - MCF7	0.793	0.00002
	Apigenin - MCF7	0.722	0.00000
	Chrysin - MCF7	0.573	0.00002
	Harmol - MCF7	0.542	0.00002
	Irinotecan - MCF7	0.508	0.00002
	Thioguanosine - MCF7	0.490	0.00002
	Bisacodyl - MCF7	0.462	0.00004
	Camptothecin - MCF7	0.452	0.00012
	Ambroxol - MCF7	−0.735	0.00070
	Heptaminol - MCF7	−0.864	0.00026
