# Supplementary material for: Multiscale Modeling of Drug-induced Effects of ReDuNing Injection on Human Disease: From Drug Molecules to Clinical Symptoms of Disease
Source: Sci Rep. 2015 May 14;5:10064. doi: 10.1038/srep10064 (PMC4431313; doi:10.1038/srep10064)
Supplement: Supplementary Information [file srep10064-s1.pdf]

Multiscale Modeling of Drug-induced Effects of RDN Injection on Human Disease: From Drug Molecules to Clinical Symptoms of Disease

Fang Luo[1], Jiangyong Gu[1], Xinzhuang Zhang[2], Lirong Chen[1†], Liang Cao[2], Na Li[2], Zhenzhong Wang[2], Wei Xiao[2†], Xiaojie Xu[1†]

1 Beijing National Laboratory for Molecular Sciences, State Key Lab of Rare Earth Material Chemistry and Applications, College of Chemistry and Molecular Engineering, Peking University, Beijing, China

2 National Key Laboratory of Pharmaceutical New Technology for Chinese Medicine, Kanion Pharmaceutical Corporation, Lianyungang, Jiangsu Province, China

† Corresponding Author

Email: xiaojxu@pku.edu.cn (Xiaojie Xu);

Email: lirongchen@pku.edu.cn (Lirong Chen);

Email: xw_kanion@163.com (Wei Xiao).

Supplementary information

---

* The first two authors contributed equally to this work.

Supplementary Table S0: Impact of the RDN ingredients on the pathway.

High Degree (31-62)		Middle degree (11-30)		Low degree (1-10)	
**Pathway Name**	**Score**	**Pathway Name**	**Score**	**Pathway Name**	**Score**
Metabolism of xenobiotics by cytochrome P450	15	Drug metabolism - cytochrome P450	10	Metabolism of xenobiotics by cytochrome P450	16
Drug metabolism - cytochrome P450	13	Serotonergic synapse	9	Drug metabolism - cytochrome P450	15
Insulin signaling pathway	11	Metabolism of xenobiotics by cytochrome P450	8	Neurotrophin signaling pathway	12
Serotonergic synapse	11	Dopaminergic synapse	7	Drug metabolism - other enzymes	12
PI3K-Akt signaling pathway	11	Glycine_ serine and threonine metabolism	6	Retinol metabolism	12
Regulation of actin cytoskeleton	10	Tryptophan metabolism	6	Colorectal cancer	11
Tuberculosis	10	Arginine and proline metabolism	5	MAPK signaling pathway	10
Peroxisome	9	Histidine metabolism	5	Steroid hormone biosynthesis	10
Steroid hormone biosynthesis	9	Toxoplasmosis	5	Focal adhesion	10
Focal adhesion	9	VEGF signaling pathway	5	FoxO signaling pathway	10
Chemokine signaling pathway	9	mTOR signaling pathway	5	Pancreatic cancer	9
Axon guidance	9	Gastric acid secretion	5	Insulin signaling pathway	9
Vascular smooth muscle contraction	9	PI3K-Akt signaling pathway	5	Cysteine and methionine metabolism	9
Endocytosis	8	Inflammatory mediator regulation of TRP channels	5	PI3K-Akt signaling pathway	9
Neurotrophin signaling pathway	8	MicroRNAs in cancer	5	Transcriptional misregulation in cancer	9
Toll-like receptor signaling pathway	8	Glioma	4	Glycine_ serine and threonine metabolism	8
Arginine and proline metabolism	8	Neurotrophin signaling pathway	4	Regulation of actin cytoskeleton	8
Tryptophan metabolism	8	MAPK signaling pathway	4	Axon guidance	8
T cell receptor signaling pathway	8	Fc gamma R-mediated phagocytosis	4	Amyotrophic lateral sclerosis (ALS)	7
Calcium signaling pathway	8	Cysteine and methionine metabolism	4	Natural killer cell mediated cytotoxicity	7

Supplementary Table S1: Network parameters of several key target proteins.

UNIPROT ID	Degree	Betweenness	UNIPROT Name
P11509	12	0.14543929	Cytochrome P450 2A6
Q6VVX0	7	0.08930768	Vitamin D 25-hydroxylase
P19801	5	0.04696212	Amiloride-sensitive amine oxidase [copper-containing]
P26358	5	0.02598916	DNA (cytosine-5)-methyltransferase 1
P27338	5	0.04885527	Amine oxidase [flavin-containing] B
P52848	5	0.04287594	Bifunctional heparan sulfate N-deacetylase/N-sulfotransferase 1
P00558	4	0.0157713	Phosphoglycerate kinase 1
P06132	4	0.03534159	Uroporphyrinogen decarboxylase
P08254	4	0.00928838	Stromelysin-1
P10635	4	0.08820364	Cytochrome P450 2D6
Q96EB6	4	0.01634897	NAD-dependent protein deacetylase sirtuin-1
Q96GA7	4	0.01556111	Serine dehydratase-like
Q9Y2Q3	4	0.03170179	Glutathione S-transferase kappa 1
O43157	3	0.01454842	Plexin-B1
O43741	3	0.00851393	5'-AMP-activated protein kinase subunit beta-2
O95749	3	0.01313714	Geranylgeranyl pyrophosphate synthase
P04040	3	0.01779917	Catalase
P05108	3	0.0227335	Cholesterol side-chain cleavage enzyme, mitochondrial
P09960	3	0.01620902	Leukotriene A-4 hydrolase
P11511	3	0.01627829	Aromatase
P12277	3	0.00610903	Creatine kinase B-type
P13569	3	0.01734233	Cystic fibrosis transmembrane conductance regulator
P21695	3	0.007109	Glycerol-3-phosphate dehydrogenase [NAD(+)], cytoplasmic
P29965	3	0.0219155	CD40 ligand
P35520	3	0.00977714	Cystathionine beta-synthase
P50336	3	0.01099991	Protoporphyrinogen oxidase
P51149	3	0.014247	Ras-related protein Rab-7a
P51449	3	0.00649119	Nuclear receptor ROR-gamma
P51857	3	0.01829734	3-oxo-5-beta-steroid 4-dehydrogenase
P60763	3	0.01975373	Ras-related C3 botulinum toxin substrate 3

Supplementary Table S2: The possible interaction network of tuberculosis.

Molecular ID	Autodock Score	Uniprot ID	Uniprot Name	Protein ID in Pathway	Pathway ID	Disease Name	Disease Symptom
UNPD149880	6.23	O60603	Toll-like receptor 2	TLR2	hsa05152	Tuberculosis	Breathing difficulty<br>Chest pain<br>Cough with mucus<br>Coughing up blood<br>Excessive sweating especially at night<br>Fatigue<br>Fever<br>Weight loss<br>Wheezing
UNPD189689	6.22	O60603	Toll-like receptor 2	TLR2			
UNPD60650	6.26	P04049	RAF proto-oncogene serine/threonine-protein kinase	Raf1			
UNPD189689	6.23	P07339	Cathepsin D	CathepsinD			
UNPD189689	6.23	P25774	Cathepsin S	CathepsinS			
RDN15	6.33	P28482	Mitogen-activated protein kinase 1	ERK1/2			
RDN1	6.5	P29460	Interleukin-12 subunit beta	IL-12			
RDN56	6.12	P51149	Ras-related protein Rab-7a	Rab7			
UNPD164733	7.14	P51149	Ras-related protein Rab-7a	Rab7			
UNPD189689	6.01	P51149	Ras-related protein Rab-7a	Rab7			
RDN47	6.08	P99999	Cytochrome c	CytC			
UNPD60430	6.08	P99999	Cytochrome c	CytC			
UNPD49205	6.29	Q13555	Calcium/calmodulin-dependent protein kinase type II subunit gamma	CAMKII			
UNPD149880	6.4	Q15399	Toll-like receptor 1	TLR1/6			
UNPD189689	6.26	Q15399	Toll-like receptor 1	TLR1/6			
UNPD51223	6.05	Q15399	Toll-like receptor 1	TLR1/6			
RDN56	6.23	Q16539	Mitogen-activated protein kinase 14	p38			
UNPD130563	6.51	Q8NEB9	Phosphatidylinositol 3-kinase catalytic subunit type 3	VPS34			
RDN20	6.24	Q9UQM7	Calcium/calmodulin-dependent protein kinase type II subunit alpha	CAMKII			
UNPD189689	6.59	Q9UQM7	Calcium/calmodulin-dependent protein kinase type II subunit alpha	CAMKII			

The red color protein ID is also in the interaction network of influenza.

Supplementary Table S3: The possible interaction network of influenza.

Molecular ID	Autodock Score	Uniprot ID	Uniprot Name	Protein ID in Pathway	Pathway ID	Disease Name	Disease Symptom
UNPD60650	6.26	P04049	RAF proto-oncogene serine/threonine-protein kinase	Raf1	hsa05164	Influenza	Body aches<br>Chills<br>Dizziness<br>Dry cough<br>Flushed face<br>Headache<br>Increased breathing symptoms<br>Lack of energy<br>Nausea and vomiting<br>Runny nose<br>Sneezing<br>Sore throat
RDN56	6.89	P05771	Protein kinase C beta type	PKCβII			
UNPD29278	6.18	P17252	Protein kinase C alpha type	PKCα			
RDN15	6.33	P28482	Mitogen-activated protein kinase 1	ERK1/2			
RDN1	6.5	P29460	Interleukin-12 subunit beta	IL-12			
UNPD130563	6.32	P29597	Non-receptor tyrosine-protein kinase TYK2	Tyk2/Jtk1			
RDNB23	6.16	P42336	Phosphatidylinositol 4,5-bisphosphate 3-kinase catalytic subunit alpha isoform	PI3K			
UNPD29278	7.03	P48736	Phosphatidylinositol 4,5-bisphosphate 3-kinase catalytic subunit gamma isoform	PI3K		Avian influenza	Cough<br>Diarrhea<br>Fever greater than 100.4°F (38°C)<br>General ill feeling(malaise)<br>Headache<br>Muscle aches<br>Runny nose<br>Sore throat<br>Trouble breathing
RDN47	6.08	P99999	Cytochrome c	CytC			
UNPD60430	6.08	P99999	Cytochrome c	CytC			
UNPD149880	6	Q02750	Dual specificity mitogen-activated protein kinase kinase 1	MEK1/2			
UNPD51223	6.92	Q02750	Dual specificity mitogen-activated protein kinase kinase 1	MEK1/2			
RDN56	6.23	Q16539	Mitogen-activated protein kinase 14	p38			
RDN21	6.69	Q9NZJ5	Eukaryotic translation initiation factor 2-alpha kinase 3	PKR			

The red color protein ID is also in the interaction network of tuberculosis.

Supplementary Table S4: The relation between several diseases and ZHENG.

Disease ID	Disease Name	Disease category	ZHENG
H00630	Disease_Rheumatoid arthritis	Autoimmune disease	Hot ZHENG
H00912	Disease_Tumor necrosis factor receptor-associated periodic syndrome (TRAPS)	Autoimmune disease	Hot ZHENG
H00001	Disease_Acute lymphoblastic leukemia (ALL) (precursor B lymphoblastic leukemia)	Cancer	Hot ZHENG
H00002	Disease_Acute lymphoblastic leukemia (ALL) (precursor T lymphoblastic leukemia)	Cancer	Hot ZHENG
H00003	Disease_Acute myeloid leukemia (AML)	Cancer	Hot ZHENG
H00004	Disease_Chronic myeloid leukemia (CML)	Cancer	Hot ZHENG
H00007	Disease_Hodgkin lymphoma	Cancer	Hot ZHENG
H00008	Disease_Burkitt lymphoma	Cancer	Hot ZHENG
H00010	Disease_Multiple myeloma	Cancer	Hot ZHENG
H00011	Disease_Lymphoplasmacytic lymphoma	Cancer	Hot ZHENG
H00013	Disease_Small cell lung cancer	Cancer	Hot ZHENG
H00020	Disease_Colorectal cancer	Cancer	Hot ZHENG
H00034	Disease_Carcinoid	Cancer	Hot ZHENG
H00295	Disease_Viral myocarditis	Cardiovascular disease	Hot ZHENG
H00465	Disease_Fragile X Syndrome	Chromosomal abnormality	Cold ZHENG
H00606	Disease_Early infantile epileptic encephalopathy	Congenital disorder; Epilepsy	Hot ZHENG
H00480	Disease_Non-syndromic X-linked mental retardation	Congenital disorder; Mental retardation	Hot ZHENG
H00577	Disease_Syndromic X-linked mental retardation with epilepsy or seizures	Congenital disorder; Mental retardation; Epilepsy	Hot ZHENG
H00478	Disease_Prader-Willi and Angelman syndromes	Developmental disorder	Hot ZHENG
H00570	Disease_Kabuki syndrome	Developmental disorder	Cold ZHENG
H00935	Disease_Cold-induced sweating syndrome (CISS) and Crisponi syndrome (CRISPS)	Developmental disorder	Hot ZHENG
H00992	Disease_Seckel syndrome	Developmental disorder	Cold ZHENG
H01288	Disease_Mosaic variegated aneuploidy (MVA) syndrome	Developmental disorder	Cold ZHENG

H00539	Disease_PTEN hamartoma tumor syndrome (PHTS)	Developmental disorder; Cancer	Cold and Hot ZHENG
H00523	Disease_Noonan syndrome and related disorders	Developmental disorder; Cardiovascular disease	Hot ZHENG
H00910	Disease_Hirschsprung disease (HD)	Developmental disorder; Digestive disease	Hot ZHENG
H00267	Disease_Holoprosencephaly (HPE)	Developmental disorder; Nervous system disease	Cold ZHENG
H00979	Disease_Caudal regression syndrome and Sirenomelia	Developmental disorder; Nervous system disease	Hot ZHENG
H00560	Disease_Pseudoxanthoma elasticum	Developmental disorder; Skin and connective tissue disease	Cold ZHENG
H00249	Disease_Thyroid hormone resistance syndrome	Endocrine disease	Hot ZHENG
H00251	Disease_Thyroid dyshormonogenesis	Endocrine disease	Cold and Hot ZHENG
H00258	Disease_Aldosterone synthase deficiency	Endocrine disease	Hot ZHENG
H00252	Disease_Congenital nephrogenic diabetes insipidus (NDI)	Endocrine disease; Kidney disease	Hot ZHENG
H00239	Disease_Bartter syndrome	Endocrine disease; Urinary system disease	Hot ZHENG
H01027	Disease_Anophthalmia and microphthalmia (A/M)	Eye disease	Cold ZHENG
H00238	Disease_Fanconi anemia	Hematologic disease	Cold ZHENG
H00081	Disease_Hashimoto's thyroiditis	Immune system disease	Hot ZHENG
H00083	Disease_Allograft rejection	Immune system disease	Hot ZHENG
H00282	Disease_Cryopyrin associated periodic syndrome (CAPS)	Immune system disease	Hot ZHENG
H00288	Disease_Familial Mediterranean fever (FMF)	Immune system disease	Hot ZHENG
H01109	Disease_Chronic mucocutaneous candidiasis (CMC)	Immune system disease	Cold ZHENG
H01232	Disease_Syndromic multisystem autoimmune disease	Immune system disease	Cold ZHENG
H00110	Disease_Cholera	Infectious disease	Hot ZHENG
H00111	Disease_Typhoid fever	Infectious disease	Hot ZHENG
H00277	Disease_Enterohemorrhagic Escherichia coli (EHEC) infection	Infectious disease	Hot ZHENG
H00299	Disease_Shigellosis	Infectious disease	Hot ZHENG
H00311	Disease_Legionellosis	Infectious disease	Hot ZHENG
H00319	Disease_Pertussis	Infectious disease	Hot ZHENG

H00342	Disease_Tuberculosis	Infectious disease	Hot ZHENG
H00357	Disease_African trypanosomiasis	Infectious disease	Hot ZHENG
H00358	Disease_Chagas disease	Infectious disease	Hot ZHENG
H00359	Disease_Leishmaniasis	Infectious disease	Hot ZHENG
H00360	Disease_Amoebiasis	Infectious disease	Hot ZHENG
H00361	Disease_Malaria	Infectious disease	Hot ZHENG
H00367	Disease_Infectious mononucleosis	Infectious disease	Hot ZHENG
H00394	Disease_Measles	Infectious disease	Hot ZHENG
H00398	Disease_Influenza	Infectious disease	Hot ZHENG
H00399	Disease_Avian influenza	Infectious disease	Hot ZHENG
H00413	Disease_Hepatitis C	Infectious disease	Hot ZHENG
H00435	Disease_Toxoplasmosis	Infectious disease	Hot ZHENG
H00069	Disease_Glycogen storage diseases (GSD)	Inherited metabolic disease	Hot ZHENG
H00118	Disease_Congenital disorders of glycosylation (CDG) type I	Inherited metabolic disease	Cold ZHENG
H00161	Disease_Smith-Lemli-Opitz syndrome	Inherited metabolic disease	Hot ZHENG
H00179	Disease_3-Hydroxy-3-methylglutaryl-CoA lyase deficiency	Inherited metabolic disease	Hot ZHENG
H01071	Disease_Acute alcohol sensitivity	Inherited metabolic disease	Hot ZHENG
H01283	Disease_Malonyl-CoA decarboxylase deficiency	Inherited metabolic disease	Hot ZHENG
H01398	Disease_Primary hyperammonemic disorders	Inherited metabolic disease	Hot ZHENG
H01400	Disease_Secondary hyperammonemia	Inherited metabolic disease	Cold ZHENG
H00881	Disease_Li-Fraumeni syndrome	Inherited metabolic disease; Cancer	Hot ZHENG
H00802	Disease_Ehlers-Danlos syndrome (EDS)	Inherited metabolic disease; Connective tissue disease	Hot ZHENG
H00216	Disease_Congenital adrenal hyperplasia (CAH)	Inherited metabolic disease; Endocrine disease	Hot ZHENG
H01199	Disease_Hyperalphalipoproteinemia	Inherited metabolic disease; Hematologic disease	Cold ZHENG
H01117	Disease_Majeed syndrome	Inherited metabolic disease; Immune system disease	Hot ZHENG

H00137	Disease_Niemann-Pick disease (NPD) typeA and B	Inherited metabolic disease; Lysosomal storage disease	Hot ZHENG
H00275	Disease_Cystinosis	Inherited metabolic disease; Lysosomal storage disease	Cold ZHENG
H00124	Disease_GM2 gangliosidoses	Inherited metabolic disease; Lysosomal storage disease; Nervous system disease	Cold ZHENG
H00424	Disease_Defects in the degradation of sphingomyelin	Inherited metabolic disease; Lysosomal storage disease; Nervous system disease	Hot ZHENG
H00426	Disease_Defects in the degradation of ganglioside	Inherited metabolic disease; Lysosomal storage disease; Nervous system disease	Cold ZHENG
H00849	Disease_Creatine deficiency syndrome	Inherited metabolic disease; Mental retardation	Hot ZHENG
H00469	Disease_Mitochondrial DNA depletion syndrome (MDS)	Inherited metabolic disease; Mitochondrial disease	Hot ZHENG
H00174	Disease_Methylmalonic aciduria (MMA)	Inherited metabolic disease; Nervous system disease	Cold ZHENG
H00692	Disease_Lowe syndrome	Inherited metabolic disease; Nervous system disease	Hot ZHENG
H00854	Disease_Wolfram syndrome (WFS)	Inherited metabolic disease; Neurodegenerative disease	Cold ZHENG
H01390	Disease_Mitochondrial neurogastrointestinal encephalomyopathy	Inherited metabolic disease; Neurodegenerative disease; Mitochondrial disease	Hot ZHENG
H00176	Disease_Adrenoleukodystrophy (ALD)	Inherited metabolic disease; Neurodegenerative disease; Peroxisomal disease	Cold ZHENG
H00202	Disease_Hepatic porphyria	Inherited metabolic disease; Skin and connective tissue disease; Liver disease	Hot ZHENG
H00756	Disease_Pitt-Hopkins syndrome	Mental retardation	Hot ZHENG
H00409	Disease_Type II diabetes mellitus	Metabolic disease; Endocrine disease	Hot ZHENG
H00410	Disease_Maturity onset diabetes of the young (MODY)	Metabolic disease; Endocrine disease	Cold ZHENG
H00937	Disease_Precocious puberty	Metabolic disease; Endocrine disease	Cold ZHENG
H00408	Disease_Type I diabetes mellitus	Metabolic disease; Immune system disease; Endocrine disease	Hot ZHENG
H00420	Disease_Familial partial lipodystrophy (FPL)	Metabolic disease; Skin and connective tissue disease	Hot ZHENG
H00652	Disease_Solitary median maxillary central incisor syndrome	Mouth and dental disease	Cold ZHENG
H00594	Disease_Distal muscular dystrophies	Musculoskeletal disease; Nervous system disease	Hot ZHENG
H00595	Disease_Myofibrillar myopathies (MFM)	Musculoskeletal disease; Nervous system disease	Hot ZHENG
H00265	Disease_Hereditary sensory and autonomic neuropathy	Nervous system disease	Hot ZHENG
H00775	Disease_Familial or sporadic hemiplegic migraine	Nervous system disease	Hot ZHENG
H00856	Disease_Distal hereditary motor neuropathies (dHMN)	Nervous system disease	Hot ZHENG

H00916	Disease_Congenital central hypoventilation syndrome (CCHS)	Nervous system disease	Hot ZHENG
H01161	Disease_Aromatic L-amino acid decarboxylase (AADC) deficiency	Nervous system disease	Hot ZHENG
H01391	Disease_Familial episodic pain syndrome (FEPS)	Nervous system disease	Hot ZHENG
H00845	Disease_Familial amyloidosis	Nervous system disease; Inherited metabolic disease	Hot ZHENG
H01118	Disease_Progressive external ophthalmoplegia (PEO)	Nervous system disease; Musculoskeletal disease; Eye disease; Inherited metabolic disease	Hot ZHENG
H00915	Disease_Tuberous sclerosis complex (TSC)	Nervous system disease; Skin and connective tissue disease	Cold ZHENG
H00057	Disease_Parkinson's disease (PD)	Neurodegenerative disease	Hot ZHENG
H00061	Disease_Prion diseases	Neurodegenerative disease	Hot ZHENG
H00455	Disease_Spinal muscular atrophy (SMA)	Neurodegenerative disease	Hot ZHENG
H00933	Disease_Hereditary pancreatitis	Pancreas disease	Hot ZHENG
H00093	Disease_Combined immunodeficiencies (CIDs)	Primary immunodeficiency	Hot ZHENG
H00107	Disease_Other well-defined immunodeficiency syndromes	Primary immunodeficiency	Cold and Hot ZHENG
H00540	Disease_Osteoporosis_ lymphedema_ anhydrotic ectodermal dysplasia with immunodeficiency (OLEDAID)	Primary immunodeficiency; Skeletal dysplasia	Hot ZHENG
H00599	Disease_46_XX disorders of sex development (Disorders related to androgen excess)	Reproductive system disease	Hot ZHENG
H00607	Disease_46_XY disorders of sex development (Disorders of gonadal development1)	Reproductive system disease	Cold and Hot ZHENG
H00608	Disease_46_XY disorders of sex development (Disorders in androgen synthesis or action)	Reproductive system disease	Cold and Hot ZHENG
H01122	Disease_Pulmonary alveolar proteinosis (PAP)	Respiratory disease	Hot ZHENG
H00894	Disease_FG syndrome (FGS)	Skeletal dysplasia; Nervous system disease	Hot ZHENG
H00651	Disease_Ectodermal dysplasia	Skin and connective tissue disease	Hot ZHENG
